# Immersive 3D Educational Contents: A Technical Note for Dental Educators

**DOI:** 10.3390/healthcare9020178

**Published:** 2021-02-07

**Authors:** Sabira Barour, Raphaël Richert, François Virard, Claudine Wulfman, Régis Iozzino, Mahmoud Elbashti, Adrien Naveau, Maxime Ducret

**Affiliations:** 1 Department de Prothèses, Université Claude Bernard Lyon 1, 69008 Lyon, France; sabira.barour@etu.univ-lyon1.fr (S.B.); raphael.richert@insa-lyon.fr (R.R.); francois.virard@univ-lyon1.fr (F.V.); iozzino.r@gmail.com (R.I.); 2 Centre de Soins Dentaires, Hospices Civils de Lyon, 69007 Lyon, France; 3 Department de Prothèses, Université de Paris, UR 4462, F-92049 Montrouge, France; claudine.wulfman@parisdescartes.fr; 4 Département d’Odontologie, AP-HP, Hôpital Henri Mondor, F-94010 Créteil, France; 5 Department of Maxillofacial Prosthetics, Tokyo Medical and Dental University, Tokyo 113-8549, Japan; mahmmfp@tmd.ac.jp; 6 Laboratory of Bioengineering of Tissues (BioTis), INSERM U1026, University of Bordeaux, 33000 Bordeaux, France; adrien.naveau@u-bordeaux.fr; 7 Unité de Parodontologie et Prothèse Dentaire, Hôpital Saint André, CHU de Bordeaux, 33000 Bordeaux, France; 8 Département de Prothèses, UFR des Sciences Odontologiques, Université de Bordeaux, 33000 Bordeaux, France; 9 Laboratoire de Biologie Tissulaire et Ingénierie Thérapeutique, UMR5305, 69367 Lyon, France

**Keywords:** 3D files, healthcare education, innovation in teaching, digital training, clinical teaching

## Abstract

Three-dimensional files featuring patients’ geometry can be obtained through common tools in dental practice, such as an intraoral scanner (IOS) or Cone Beam Computed Tomography (CBCT). The use of 3D files in medical education is promoted, but only few methodologies were reported due to the lack of ease to use and accessible protocols for educators. The aim of this work was to present innovative and accessible methodologies to create 3D files in dental education. The first step requires the definition of the educational outcomes and the situations of interest. The second step relies on the use of IOS and CBCT to digitize the content. The last “post-treatment” steps involve free software for analysis of quality, re-meshing and simplifying the file in accordance with the desired educational activity. Several examples of educational activities using 3D files are illustrated in dental education and discussed. Three-dimensional files open up many accessible applications for a dental educator, but further investigations are required to develop collaborative tools and prevent educational inequalities between establishments.

## 1. Introduction

Historically, 3D files are considered as relatively inaccessible educational tools, mainly because they required 3D designers and significant technological and human resources. However, in the medical field, imaging devices are already well developed, such as in dentistry for many clinical applications [1,2]. These devices could also open up many applications through the use of digitized 3D files [3,4,5,6,7]. They were reported to improve the capacity of medical and dental students of learning disciplines such as anatomy [4,7,8]. Moreover, the development of educational platforms and hybrid teaching is especially useful in these times of COVID-19, as they increase the educational possibilities, that include learning of spatial ability, interactivity, critical thinking and decision-making [8,9]. However, the available resources for teaching are often limited in dental schools, and the lack of ease to use and accessible protocols for educators complicate the production of 3D education content [7,10]. Therefore, an alternative proposal appears necessary for dental educators to create, visualize and interact with 3D educational contents in a format and a weight that allows for use on the institutional educational platform or in additional software.

The aim of this article was to present innovative and accessible methodologies to create 3D educational files in dental education with common imaging devices such as intraoral scanner (IOS) or Cone Beam Computed Tomography (CBCT), and using free software.

## 2. Methodology

### 2.1. Alignment of Educational Objective to Educational Content

**Figure 1:**
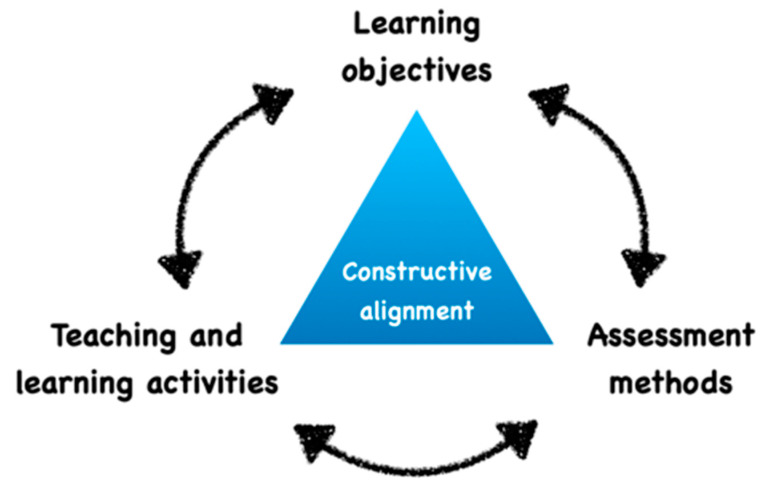
Concept of educational alignment between the objectives, teaching context and activities.

The first step requires us to clarify the educational objectives and the method of evaluation. Indeed, the teacher has to carefully organize and align all the objectives, teaching context and activities [11,12] (Figure 1). For example, does the educator plan to measure “how a student is able to estimate the volume of a decay”, “how a student is able to visualize the design of the future endodontic access cavity” or “how student is able to indicate the required treatment or to select the adapted instrument”? These three questions could be asked using similar 3D files, but they actually measure completely different capacities.

### 2.2. Create or Collect the Situation

**Figure 2:**
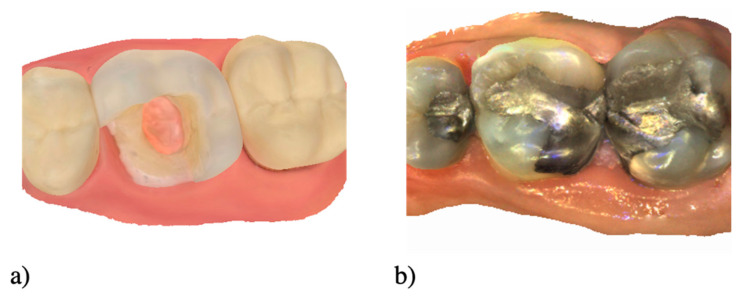
Example of two 3D educational contents using IOS with artificial teeth (**a**) and a clinical situation (**b**).

Once the educational alignment has been thought through, the next step is to create or collect 3D situations. It could be simple or complex cases clinically encountered, or artificial situations from educational materials such as typodont teeth, or from extracted teeth [13] (Figure 2). Finally, all these decisions will drive the choice of whether to digitize with IOS or the CBCT.

### 2.3. Scan with an IOS

Clinical situations can be digitized with an IOS to obtain three-dimensional (3D) files. The raw file collected by the IOS can either be black and white triangular mesh (standard triangle language (STL), or triangles with additional information about color and texture (.ply and .obj). The areas of interest for the educational tool can be scanned in high dimension (HD) in order to obtain highest definition and more details. Immersivity can also be improved using operating field and materials, for example using rubber dam or occlusal contact with articulating paper.

### 2.4. Scan with a CBCT

**Figure 3:**
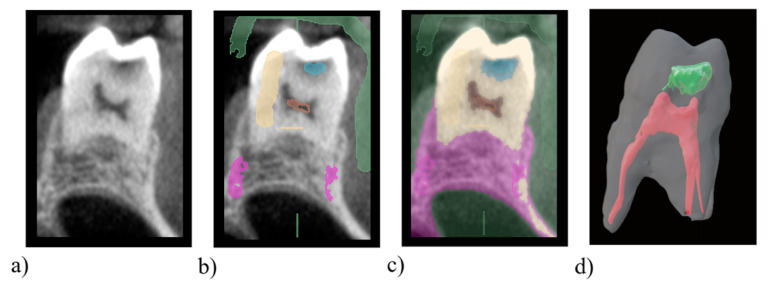
Conception of an educational model with carious process using a free software 3DSlicer. (**a**) 3D image from a cone beam computed tomography of a maxillary molar presenting a cavity. (**b**) Attribution of pixel labels for air (green), dentin (yellow), pulp (red), cavity (blue) and bone (purple) using the “paint” tool on the 3D image. (**c**) Segmentation of the 3D image using the “Grow from seeds” tool. (**d**) Export of the pulp, cavity and tooth STL models and visualization in the software Blender using transparent labels.

Settings of CBCT are first optimized according to the object size. When selecting the clinical situation and in order to facilitate image interpretation, images deformed by artefacts, for example if large volumes of metallic materials are present, should be rejected. The segmentation could be conducted using different strategies such as gray-level threshold, a region-based technique or a deep learning method [14,15]. The different dental structures can be marked on the 3D image using labels and segmented with a free software depending on their Hounsfield units (3D Slicer, ITK-SNAP). The 3D-labelled image is then meshed and exported as an STL file for further educational activities (Figure 3).

### 2.5. Post Treatement

#### 2.5.1. Quality Analysis of 3D File

**Figure 4:**
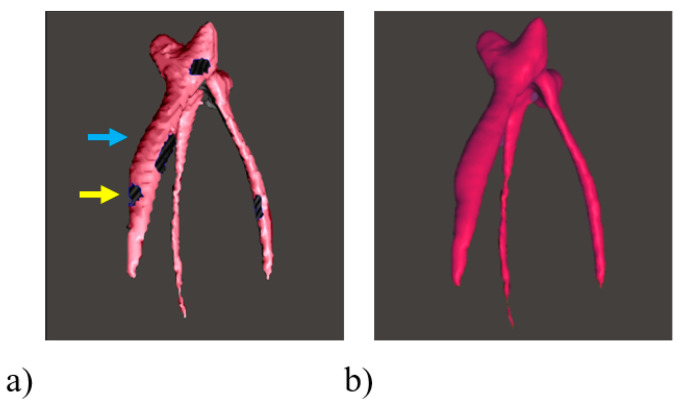
Cleaning and repairing the 3D file of a segmented pulp using the free software Meshmixer (Autodesk, San Rafael, EU). (**a**) STL presenting holes (yellow arrow) and stair-steps (blue arrow) due to the resolution of the cone beam computed tomography, could be automatically smoothed and repaired using “Smooth” and “Inspector” tools in the software (**b**).

The 3D file obtained from IOS or CBCT corresponds to a point cloud forming triangles. This mesh corresponds to a network of different density and quality of triangles that can be analyzed using free software such as Meshlab (ISTI and CNR, Rome, Italy) or Blender (Blender Foundation, Amsterdam, Netherlands). An ideal mesh corresponds to fairly equilateral, or at least fairly identical, triangles. In order to be as fair as possible, it could be suggested that one removes the color during the analysis of the 3D file [6]. The 3D file could also be repaired, closed or smoothed to improve the rendering using free software such as Meshmixer (Autodesk, San Rafael, EU) (Figure 4).

#### 2.5.2. Re-Meshing and Simplification

**Figure 5:**
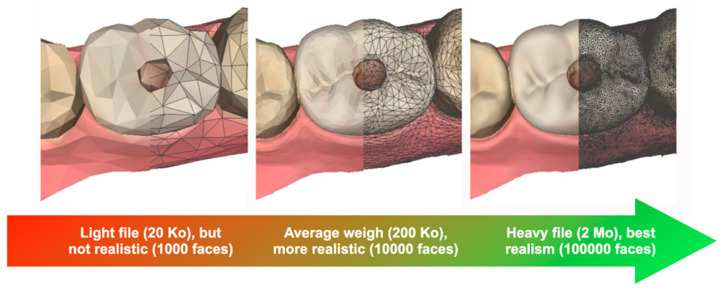
Impact of mesh density (number of faces) on file size and realism.

Redefining or reducing the size of the 3D file facilitates its integration into the educational software. Indeed, a large file has a large definition and therefore a greater weight (Figure 5). 

This high definition is less useful for an educational model, where immersivity is enhanced by a fast-loading time. Once again, free software (Meshlab or Blender) can be used for this step. Finally, the 3D file could also be prepared and transformed in 3D PDF (Adobe, San josé, EU) [7] to improve visualization and enhance their spatial ability, or for 3D printing, using a “model builder” tools [16]. Semi-automatic tools are generally offered by most of the dental Computer-Aided Design (CAD) softwares, but it could also be done manually using free software such as Blender.

## 3. Discussion

Digital technologies open up the possibility to improve dental education. A proposition is made here to help educators easily prepare 3D files. However, several questions regarding the educational outcomes and the way 3D files will be used remain.

**Figure 6:**
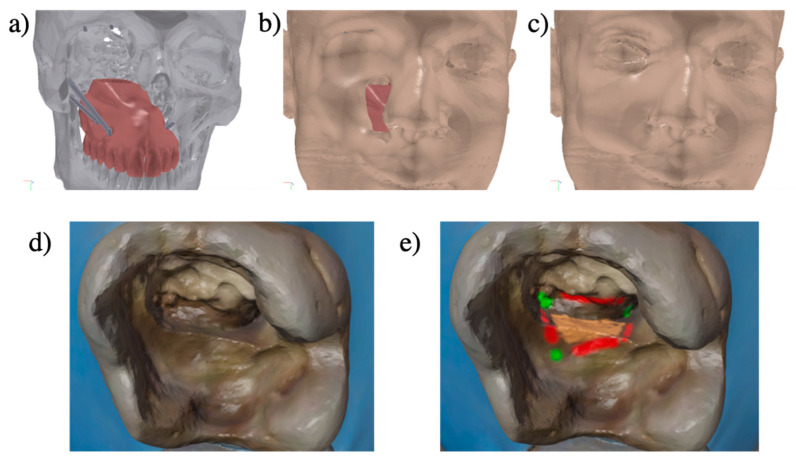
Two innovative activities using 3D files. 3D-PDF format could be used to offer simplified visualization of maxillofacial prosthetics concepts to dental students, by interacting with different volumes, or using preselected views (**a**–**c**). An incorrect 3D endodontic cavity (**d**) could be used to ask student to: -determine the root canal (green), -the form of the future endodontic access cavity (red) and the corrections to realize (orange), using 3D “painting” tools (**e**) (here using the Vertex paint mode of Blender).

The most challenging points for universities to integrate 3D files will be to reach a consensus regarding the outcomes of these tools, and define a way to measure their effectiveness [17]. These tools are not intended to replace all forms of pedagogy validated for decades. Yet they may lever the current limitations of video or 2D images in lack of immersivity or complexity for several students to visualize and transform 2D information in 3D [18]. Indeed, faced with this difficulty of 3D visualization, several authors already proposed 3D files in prosthetic dentistry, endodontic anatomy or for presenting maxillofacial prosthetics concepts [7,19,20] (Figure 6a–c). Indeed, the capacity of 3D visualization could be predictive of the learning of complex tasks [21]. Recently, a mobile application was developed to use 3D visualization to learn and assess the decision making in restorative dentistry [9].

A large number of other applications based on IOS and 3D files to improve learning and assessment of students during tooth preparation is possible [22,23,24]. It was also proposed to use 3D files as 3D printed educational tools in medical education [25]. Authors reported that it could be useful also in dental education, to teach for example endodontology [26]. A recent study also reported the use of 3D files based on real patient situation to teach dental surgery [27]. Moreover, these files can be easily integrated into preclinical simulation using haptic or virtual reality simulators, which is regarded with interest in dental education [7,28,29].

The generalization of 3D files should be approached with caution, because it is still emerging in the field of education. The prime limitation in following such a protocol is the need for educators with experience in non-dental software, that may impose collaborations with other universities or training. Moreover, 3D files can sometimes lack an entertaining aspect and be too complex for students to manipulate [30], and therefore require the development of more user-friendly interphase. As the resources for the development of these educative tools are not always known, a dental faculty network, open-access publications or educational congresses could be relevant strategies to share tips and tools, such as 3D PDF files [7], free software to interact and paint on 3D files (Paint 3D, Microsoft, Redmond, EU) or Blender (Figure 6d,e), free web-based platforms (Metaciel, Université Clermont Auvergne, Clermont-Ferrand, France) [31], or open source codes of applications [9]. However, these practices are still recent, and additional studies will be necessary to deeply evaluate how these tools will help dental educators to teach and students to achieve their educational goals.

## 4. Conclusions

This article presented digital methodologies, tips and tools to create immersive 3D contents for dental education using IOS or CBCT and free software. These procedures open up many applications and possible educational collaborations for dental educators. Further comparisons with current practices will be required to identify and select 3D tools that really improve teaching and learning.

## Data Availability

Not applicable.
